# Physician’s sociodemographic profile and distribution across public and private health care: an insight into physicians’ dual practice in Brazil

**DOI:** 10.1186/s12913-018-3076-z

**Published:** 2018-04-23

**Authors:** Bruno Alonso Miotto, Aline Gil Alves Guilloux, Alex Jones Flores Cassenote, Giulia Marcelino Mainardi, Giuliano Russo, Mário César Scheffer

**Affiliations:** 1 Department of Preventive Medicine, Faculdade de Medicina, Universidade de São Paulo, Doutor Arnaldo Avenue, nº 455, São Paulo, SP 01246-903 Brazil; 2 Centre for Primary Care and Public Health, Queen Mary University of London, 58 Turner Street, London, E1 2AB UK

**Keywords:** Dual practice, Private practice, Public practice, Physicians, Brazilian healthcare system, Physician behaviour, Human resources for health

## Abstract

**Background:**

The intertwined relation between public and private care in Brazil is reshaping the medical profession, possibly affecting the distribution and profile of the country’s medical workforce. Physicians’ simultaneous engagement in public and private services is a common and unregulated practice in Brazil, but the influence played by contextual factors and personal characteristics over dual practice engagement are still poorly understood. This study aimed at exploring the sociodemographic profile of Brazilian physicians to shed light on the links between their personal characteristics and their distribution across public and private services.

**Methods:**

A nation-wide cross-sectional study using primary data was conducted in 2014. A representative sample size of 2400 physicians was calculated based  on the National Council of Medicine database registries; telephone interviews were conducted to explore physicians’ sociodemographic characteristics and their engagement with public and private services.

**Results:**

From the 2400 physicians included, 51.45% were currently working in both the public and private services, while 26.95% and 21.58% were working exclusively in the private and public sectors, respectively. Public sector physicians were found to be younger (PR 0.84 [0.68–0.89]; PR 0.47 [0.38–0.56]), less experienced (PR 0.78 [0.73–0.94]; PR 0.44 [0.36–0.53]) and predominantly female (PR 0.79 [0.71–0.88]; PR 0.68 [0.6–0.78]) when compared to dual and private practitioners; their income was substantially lower than those working exclusively for the private (PR 0.58 [0.48–0.69]) and mixed sectors (PR 0.31 [0.25–0.37]). Conversely, physicians from the private sector were found to be typically senior (PR 1.96 [1.58–2.43]), specialized (PR 1.29 [1.17–1.42]) and male (PR 1.35 [1.21–1.51]), often working less than 20 h per week (PR 2.04 [1.4–2.96]). Dual practitioners were mostly middle-aged (PR 1.3 [1.16–1.45]), male specialists with 10 to 30 years of medical practice (PR 1.23 [1.11–1.37]).

**Conclusion:**

The study shows that more than half of Brazilian physicians currently engage with dual practice, while only one fifth dedicate exclusively to public services, highlighting also substantial differences in socio-demographic and work-related characteristics between public, private and dual-practitioners. These results are consistent with the international literature suggesting that physicians’ sociodemographic characteristics can help predict dual practice forms and prevalence in a country.

**Electronic supplementary material:**

The online version of this article (10.1186/s12913-018-3076-z) contains supplementary material, which is available to authorized users.

## Background

Physicians’ concurring engagement in multiple public and private sector clinical activities, a modality of practice often referred as dual practice, has been identified as a widespread phenomenon across different national health systems [1–3]. Multiple negative and positive aspects regarding dual practice have been highlighted in the public health and health economics literature [2, 3], and although few studies have pointed out at some positive effects of holding multiple job positions over healthcare provision [4], mostly attributed to savings on public expenditure and the possibility of retaining specialist doctors in the public sector, it is a widespread concern that this practice could potentially reduce the accessibility and quality of care in public health systems in low- and middle-income countries [5, 6]. Previous studies [6, 7] indicate that the comparatively better conditions offered by the private sector, such as higher remuneration and more flexibility of working hours, would promote physician’s shirking from public duties to dedicate time to more lucrative private activities, diversion of patients from public to private facilities, and the use of public resources to support privately provided services [8, 9].

Although the evidence base on the effects and motivations of physicians’ dual practice is scant, some have highlighted that it is likely to represent a formidable hurdle for the achievement of Universal Health Coverage and Sustainable Development Goals [5]. For such reasons, understanding dual practice has been considered one of the top priorities for human resources for health research in low- and middle-income countries [5, 10]. The extent and forms of such practice across different national health systems seem to depend primarily on how local physicians interact within the public and private spheres, local labour markets, and on how local legislation regulates medical healthcare services [5, 11]. Some studies have shown that physicians’ personal characteristics such as age, sex, specialty and years of practice, can predict their engagement in dual practice [12].

The present paper aims at exploring forms and extent of different medical practice modalities in Brazil, including dual practice. Brazil is one of world’s most populated countries, operating the largest public health system worldwide, where public and private funds and services are almost inextricably mixed to provide care for the country’s very diverse population.

Brazil is a middle-income Federative Republic composed of 26 states and one Federal District, with a population of approximately 206,081,432 inhabitants. The country has a Human Development Index of 0.755 and a gross domestic product (GDP) of US$ 11,067 per capita [13]. It is marked by profound health disparities [14], and despite the major advances achieved towards the decrease of infant mortality rates and infectious diseases control [15], Brazil still struggles to overcome critical distribution inequalities between public and private services [16].

Since 1988, the country has implemented a tax-funded Unified Health System (SUS, from its Portuguese acronym), providing primary health care and some tertiary care services to its population free  at the point of delivery. While the majority of the Brazilian population depends exclusively on public healthcare provided by the SUS (approximately 75% of the country’s population), private expenditures still represents 54% of total health expenditures, with 39% of out-of pocket payments [17]. Some authors have therefore defined Brazil’s health system as a mixed system predominantly funded by private resources [14].

However, no clear-cut distinction exists between private and public sectors in Brazil, as (a) for certain medical acts the public sector can purchase services from both private for-profit and private not-for-profit organizations; (b) public facilities can be used to provide private services; (c) public facilities can be managed by private companies; and (d) public funds may be allocated to both private and public institutions [18, 19].

Similarly to what observed in other high-, middle- and low-income countries [4, 20, 21], the medical profession in Brazil is marked by the coexistence of multiple job affiliations and possibilities of insertion in the health system [22], including working part-time jobs in both public and private services. Previous studies have shown substantial differences in the availability and supply of physicians for private plan customers and those depending exclusively on the SUS, and a increasingly proportion of job positions for the medical workforce has been provided by the private sector [23].

Over the last 30 years, a number of policies have been implemented in Brazil to attenuate physician’s regional and public/private distribution disparities. More recently, the Federal Government has implemented the *Mais Médicos* programme (More Doctors Law; October 22, 2013), in order to promote the retention of health workers in unassisted areas and the opening of several new medical schools and internship programs. The programme also addresses educational policies towards changes in medical school curriculum, which should in theory provide an incentive to new doctors to take up positions in priority primary care services. The Law, however, does not establish any restrictions for holding simultaneous public and private job positions, and there are no current policies to regulate such practice.

Even though dual practice is a common and unrestricted activity in Brazil, the proportion and the sociodemographic profile of Brazilian physicians working as dual practitioners have been conspicuously under-researched. Most available information addressing the profile of the medical workforce in Brazil is currently based on data provided by local medical associations and/or national public access databases; medical registries from these repositories are often incomplete, inaccurate or missing, with conflicting information across different sources. The evaluation of the extent of dual practice and physician’s demographic characteristics, such as age, region, gender, income, years of medical practice, public or private education and weekly workload, may help to better understand the impact of physicians’ multiple job-holding over healthcare accessibility in Brazil.

The present study presents the findings from the first representative nation-wide survey of the Brazilian medical workforce. Primary data was used to establish linkages between physician’s sociodemographic and work-related characteristics to their engagement with public and private services in Brazil, with a specific reference to those engaging in dual practice. The ultimate goal of this work is to contribute to the existing debate on the determinants and consequences of physicians’ dual practice in low- and middle-income settings.

## Methods

### Study and sample design

**Fig. 1:**
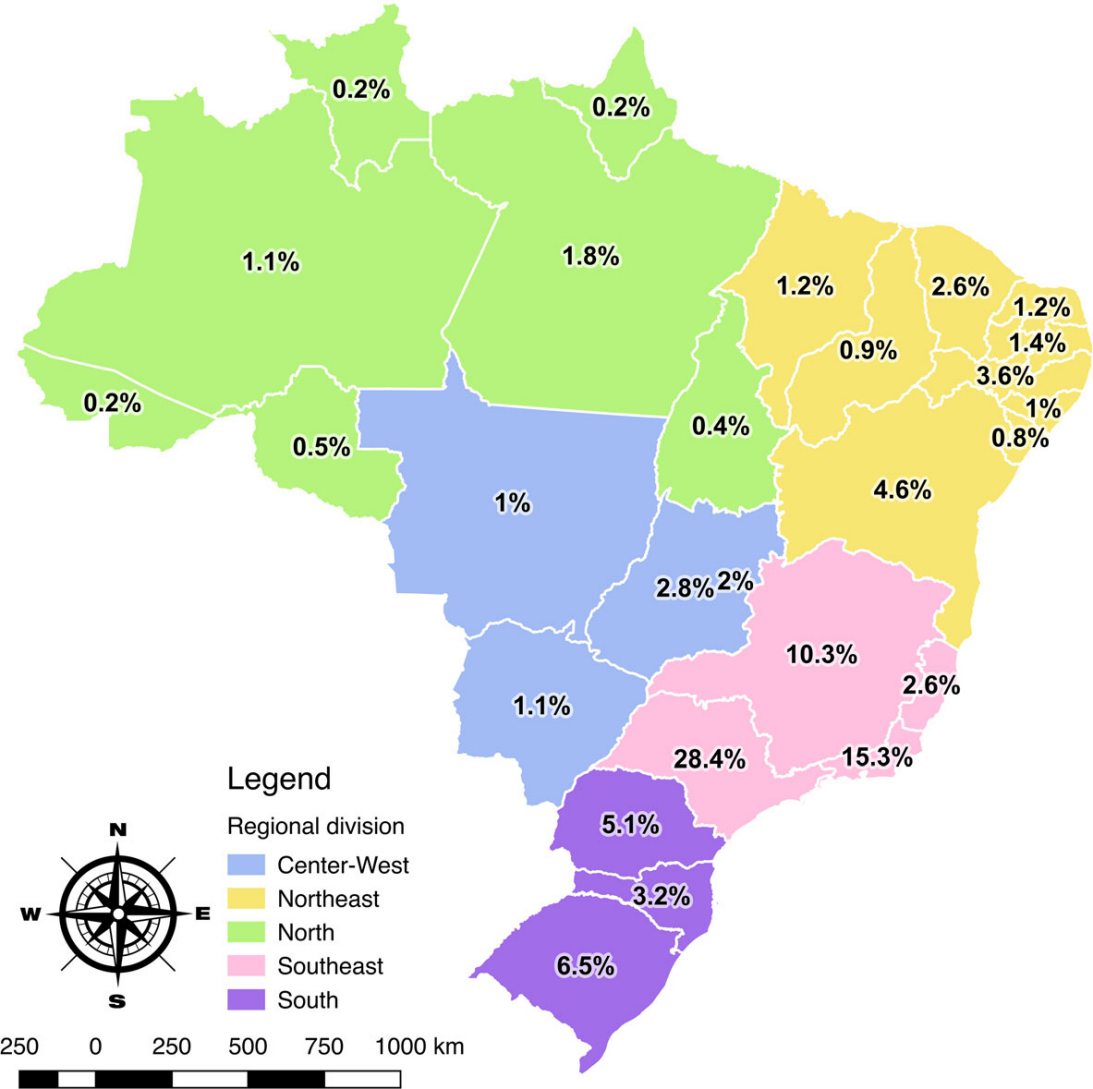
Proportional stratified sampling according to the population from each Brazilian region; all regions were considered statistical stratum. Source: Miotto et al. 2018

A nation-wide cross-sectional study including 2400 physicians was conducted in 2014. Sample size was calculated based on a total of 399,692 active medical registries from the National Medical Council Medicine database (CFM – Conselho Federal de Medicina), using a 95% confidence level with 5% margin of error and statistical power of 80% [22]. Proportional stratified sampling was drawn according to the population from each Brazilian region (Northern, Northeastern, Southeastern, Southern and Center-western regions), which were considered as statistical stratum (Fig. 1). Within each stratum, the physician’s distribution for gender, age, state and local of address (capital or countryside) were preserved.

Substitution was carried out exclusively in cases of unsuccessful contact or refusal to participate in our survey; 2,400 physicians were randomly selected and five substitutions were identified for each individual. Substitution sampling followed the same stratification criteria used for the initial sample calculation. We controlled sample replacements for state, region, sex and age, meaning that every physician who refused to participate was replaced by an individual with the same characteristics to avoid selection bias.

Primary data were collected via a telephone survey approach by 14 data collectors, including one field coordinator, 11 experienced interviewers and two professionals responsible for checking missing data. Sample size calculation, sample selection, questionnaire design, substitution control, database assembly and data analysis were performed by the authors. Data collection was carried out by the Datafolha Research Institute under financial support and supervision of the authors’ research institution. The interviews consisted of a 30-min oral questionnaire, containing 30 questions ranging from multiple, closed questions to interdependently concatenated and semi-opened questions, as presented in an additional file (see Additional file 1). Three senior researchers from the medical demography field previously piloted and calibrated the questionnaire with 30 interviewees to estimate the reposition rate. Reproducibility was tested by sampling a random sample after the field collection and repeating the interview, resulting in 100% agreement.

The independent variables used were divided into two groups: (1) sociodemographic characteristics and (2) medical employment characteristics. For the purpose of international comparison, the values in Brazilian currency (R$) were converted into US dollars (US$) based on the exchange value of R$2.0742 for US$1 (average exchange rate for 2013). Only income values obtained from activities related to the medical profession were considered. The dependent variable ‘mode of practice’ was divided into three categories: (a) working exclusively as a public practitioner, (b) working exclusively as a private practitioner and (c) working simultaneously in public and private practices. For the present study, the public sector services were defined as “those services offered free-of-charges by the SUS, funded through general taxation and social contribution”. Private services were considered “those offered through private health insurances funded through private and employer’s contributions, or out-of-pocket expenditures”.

### Statistical analysis

The differences in sociodemographic/work-related characteristics and prevalence rates (PR) between physicians were established using prevalence ratio and 95% confidence intervals. The selected variables were initially studied with frequency analysis including 95% confidence intervals (95%CI) estimated from 1000 bootstrap samples. Data was stratified according to the nature of the services (public, private or dual practice). Unadjusted (crude) prevalence ratio for dual practitioners [(PR (dual practitioners/ public practitioners)] and private practitioners [(PR (private practitioners/public practitioners)], including confidence intervals (95%CI), were used to evaluate influences of the independent variables over the dependent variables. All statistical analysis was performed in IBM SPSS Statistics version 21.

This study was reviewed and approved by the Medical School Research Ethics Committee from São Paulo University (Protocol Number 79.424), in accordance with Brazilian and international regulations for research with human subjects. All the physicians interviewed gave their informed verbal consent before participating in the study.

## Results

**Fig. 2:**
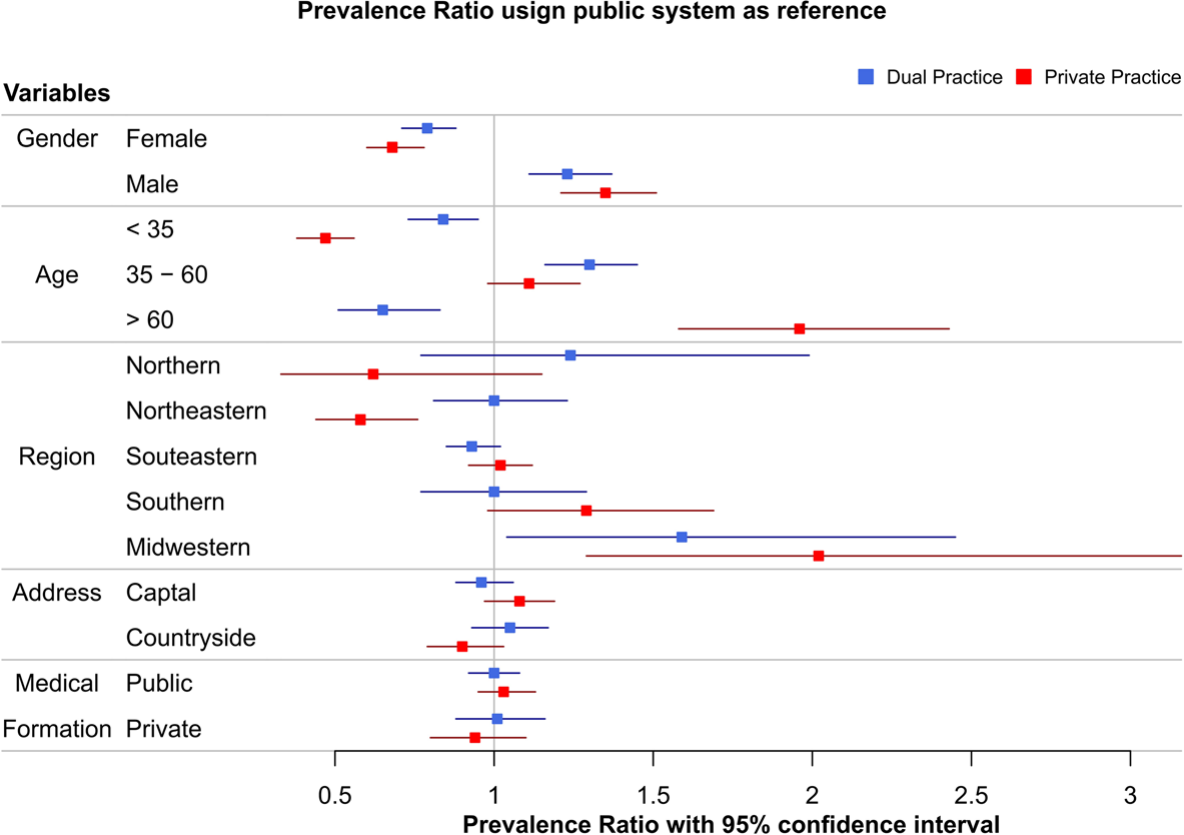
Forest plot of PR and PR 95%CI for sociodemographic characteristics of physicians working as dual and private practitioners, taking public practitioners as reference. Source: Miotto et al. 2018

**Fig. 3:**
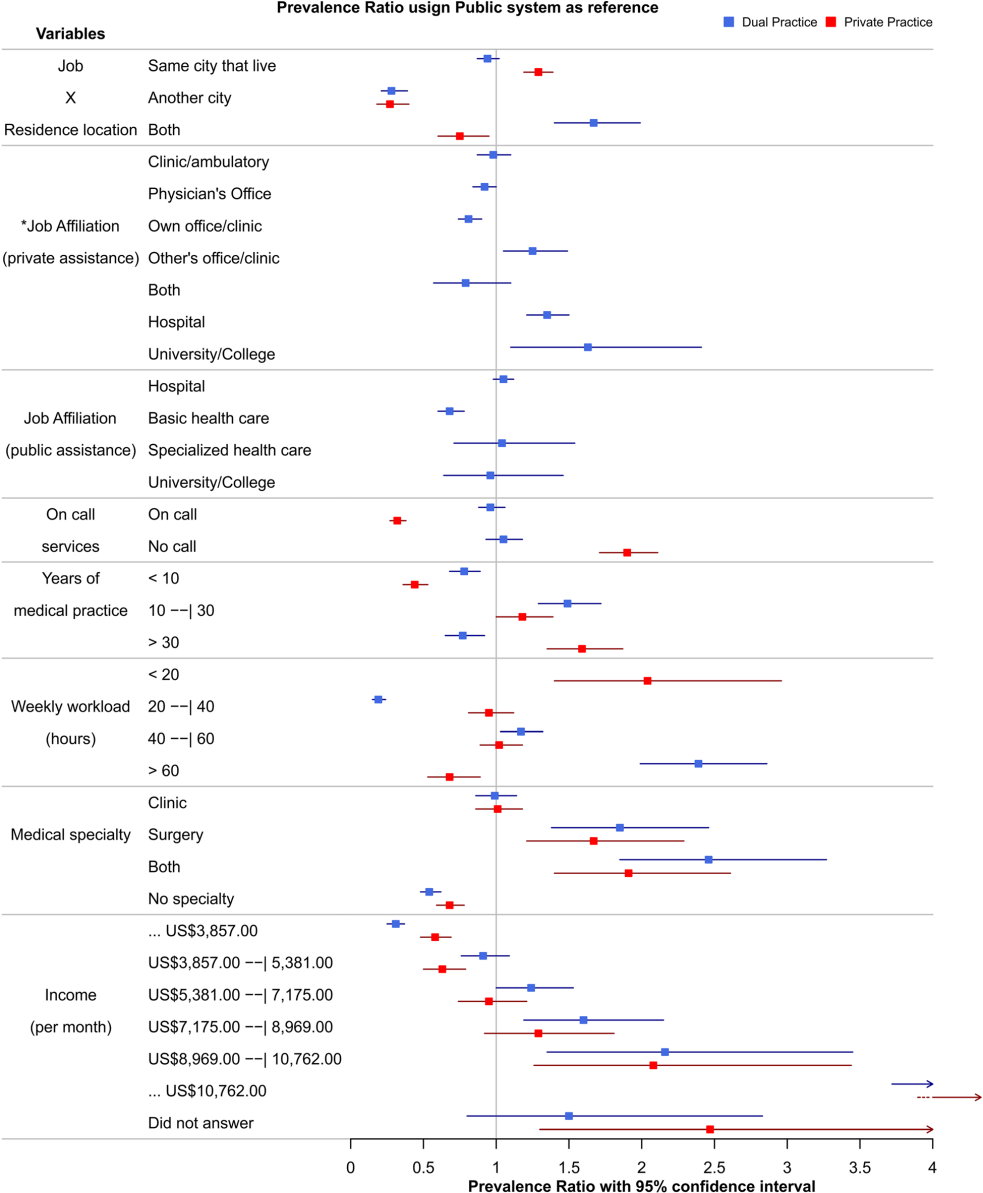
Forest plot of PR and PR 95%CI for work-related characteristics of physicians working as dual and private practitioners, taking public practitioners as reference. * This variable had the private system as comparison basis, as doctors in the public system do not have private practice activities. Source: Miotto et al. 2018

Of the 2400 physicians included in the study, 51.45% (*n* = 1235; 95% CI 49.42–53.42) declared   working as dual practitioners, while 26.95% (*n* = 647; 95% CI 25.17–28.79) and 21.58% (*n* = 518; 95% CI 19.88–23.33) were working exclusively as private and public practitioners, respectively (Additional files 2 and 3). All the prevalence rates and PR confidence intervals addressing sociodemographic and work-related differences between public, private and dual practitioners described in the text were calculated taking the public practitioners as reference (Figs. 2 and Fig. 3).

In the study, 57.5% were males and 42.5% were females (95% CI 55.4%–59.6%; 40.4%–44.6%), in agreement to the general distribution of physicians in Brazil. Most physicians working exclusively as private practitioners or as dual practitioners were males (64 and 58.3%, respectively – 95% CI 60.4%–67.4%; 55.5%–61.1%). Prevalence of male physicians working exclusively as private practitioners was 35% higher in comparison to public assistance physicians (PR 95% CI 1.21–1.51). Conversely, female physicians were 46,3 and 26,4% more prevalent among public practitioners rather than private and dual practitioners, respectively (PR 95% CI 0.6–0.78; 0.71–0.88).

Younger physicians (less than 35 years of age) were 114.7% and 19.5% more prevalent among public physicians than private and dual practitioners, respectively (PR 95% CI 0.38–0.56; 0.73–0.95). Physicians with ages ranging from 35 to 60 years were 30% more prevalent in the dual practice category in relation to the public practice category (PR 95% CI 1.16–1.45), while no significant age difference was found in the prevalence rates between public and private physicians. Physicians above 60 years of age were 96% more prevalent among private practitioners than physicians delivering public services (PR 95% CI 1.58–2.43), whereas senior dual practitioners were 54.4% less prevalent as compared to public practitioners (PR 95% CI 0.51–0.83).

Most physicians included in the study were located at the Southeast region (56%; 95% CI 54%–58%) followed by the Northeast (17.3%; 95% CI 15.7%–18.7%), South (14.8%; 95% CI 13.5%–16.2%),  Central-western (7.6%; 95% CI 6.6%–8.7%) and North regions (4.3%; 95% CI 3.4%–5.2%), in agreement to the general distribution of physicians in Brazil. Despite the marked inequality of physician’s distribution across the country, only two regions showed differences in prevalence rates between public, dual and private practice: the northeast region presented higher prevalence of physicians working as public practitioners (72,8%; PR 95% CI 0.44–0.76) when compared to private practitioners, as opposed to the Central-western region, where dual and private practitioners were respectively 59% and 102% more prevalent than public practitioners (PR 95% CI 1.04–2.45 and 1.29–3.16).

Most physicians (64.1%; 95% CI 62.1%–65.9%) reported working at their city of residence (same city). Only 7% (95% CI 6.1%–8.1%) reported working exclusively in a city that they were not living in (different city), while 28.9% (95% CI 27.1%–30.8%) occupied job positions located both at their cities of residence, but also in another city (both). Physicians working in the same city they live were 29% more prevalent among private practitioners than physicians working in public services (PR 95% CI 1.19–1.39). Conversely, the prevalence of doctors working exclusively in a different city in the public sector was 251.4 and 274.7% higher than among dual and private practitioners, respectively (PR 95% CI 0.21–0.39 and 0.18–0.4). Physicians working in both same and different cities were 67% more prevalent among dual practitioners in comparison to physicians delivering public services (PR 95% CI 1.4–1.99).

Most physicians providing private services (including dual or private practitioners) were working at medical clinics and private hospitals, representing 78.2% (95% CI 74.3%–82.2%) of the interviewees; 31.1% reported working at private clinics/ambulatories (95% CI 29.3%–32.8%), and only 5.3% were currently working at private universities (95% CI 4.3%–5.2%).

Physicians providing public services (including dual practitioners and doctors dedicated exclusively to public practice) were more frequently registered at public hospitals (51.5%; 95% CI 49.5%–53.5%), followed by primary care (23.5%; 95% CI 22%–25.2%), specialized care (4.8%; 95% CI 3.9%–5.7%) and public college/university institutions (4.1%; 95% CI 3.3%–5%). Primary care physicians were 46.2% more prevalent among doctors exclusively working as public practitioners than dual practitioners (PR 95% CI 0.6–0.78).

Most doctors reported not working in on-call services (54.5%; 95% CI 52.5%–56.5%). Public practice physicians that provided on-call services were 212.3% more prevalent as compared to private service physicians (PR 95% CI 0.27–0.38). Prevalence of on-call services was similar between public and dual practice physicians.

In relation to the years of medical practice, doctors with less than 10 years of medical practice represented 29.5% (95% CI 27.5%–31.4%), practically the same proportion of physicians with more than 30 years of practice (29.9%; 95% CI 28%–31.8%). Physicians with less than 10 years of practice were concentrated in public services (28% and 127.9% more prevalent than dual and private practitioners, respectively; PR 95% CI 0.68–0.89; 0.36–0.53). Physicians with 10 to 30 years of practice were 49% more prevalent among dual practitioners as compared to their public sector peers (PR 95% CI 1.29–1.72). Physicians with more than 30 years of practice were concentrated among private practitioners (59% more prevalent than the public service physicians; PR 95% CI 1.35–1.87). Nevertheless, physicians with more than 30 years of practice were 77% more prevalent among public practitioners than dual practitioners (PR 95% CI 0.65–0.92).

Weekly workload ranging from 40 to 60 h was reported by 43,1% of the interviewees (95% CI 41.1%–45%); 32.4% reported working more than 60 h per week (95% CI 30.5%–34.2%). Nearly one fifth (19,4%; 95% CI 18%–20.9%) of physicians reported a weekly workload of 20 to 40 h and only 5.2% reported working less than 20 h per week (95% CI 4.3%–6.2%).

Distribution of physician’s workload according to their mode of practice revealed that private practitioners were over-represented in the 20 h per week category (prevalence rate 104% greater than public practitioners; PR 95% CI 1.4–2.96), showing also no dual practitioners in this category. Few dual practitioners dedicate 20 to 40 working hours per week, in contrast to physicians from public services, which are 424.5% more prevalent than doctors dedicated to dual practice when considering this category/interval (PR 95% CI 0.15–0.24). Dual practitioners, however, are more prevalent in the 40 to 60 working hours category (17%; PR 95% CI 1.3–1.32), showing also marked prevalence among physicians working more than 60 h per week as compared to public practitioners (139%; PR 95% CI 1.99–2.86). Besides dual practitioners had presented the most extended weekly workloads, public service physicians working more than 60 h per week were 46.2% more prevalent than private practitioners (PR 95% CI 0.53–0.89).

Of all physicians included in the study, 68% held a specialization (95% CI 66.3%–69.8%). Specialists were more prevalent among dual and private practitioners than physicians delivering public assistance (41 and 29%, respectively; PR 95% CI 1.29–1.54; 1.17–1.42). Physicians holding surgery specialties were 85% and 67% more prevalent among dual and private practitioners as compared to the public practitioners, respectively (PR 95% CI 1.38–2.46; 1.21–2.29). These differences became more evident across the categories when surgery and general clinical specialties were considered (146% and 91% more prevalent among dual and private practitioners rather than public practitioners – PR 95% CI 1.85–3.27%; 1.40–2.61).

More than half of the physicians surveyed (55.5%; 95% CI 51.0%–60.0%) reported wages ranging from US$ 3857.00 to US$8969.00. Lower incomes (below US$ 3857.00) were reported by 20% of the interviewees (95% CI 18.4%–21.7%), whereas the highest income values (more than US$8969.00) were reported by 20.7% of physicians (95% CI 18.3%–23.1%).

Distribution of physician’s income across the sectors revealed marked differences between the categories; physicians earning less than US$ 3857.00 were 226,8% (PR 95% CI 0.25–0.37) and 73,6% (PR 95% CI 0.48–0.69) more prevalent among public practitioners in relation to dual and private practice physicians, respectively. Contrastingly, physicians with monthly wages ranging from US$8969.00 to US$10,762.00 were 116 and 108% more prevalent among dual and private practitioners, respectively, as compared to public practice physicians (PR 95% CI 1.35–3.45 and 1.26–3.44). The same distortions were intensified among physicians earning more than US$10,762.00: professionals who fell into this category were 661 and 807% more prevalent among dual and private practitioners, respectively, in relation to physicians providing public medical services (PR 95% CI 3.72–11.73; 4.51–14.44).

## Discussion

This study shows that more than half of Brazilian physicians engage with dual practice, while nearly one fifth are employed exclusively as public practitioners. Dual practitioners are mostly middle-aged male professionals with 10 to 30 years of medical practice, working in medical offices at state capitals of the wealthiest regions of Brazil. Most dual practitioners hold a specialisation and undertake the heaviest workloads, with their income ranging from US$ 3857.00 to US$8969.00. On the other hand, doctors working exclusively in the public sector are predominantly female, younger, not specialized and less experienced physicians, and their income are substantially lower compared to dual and private practitioners. Physicians dedicated exclusively to private services were found to be senior, specialized, male doctors that often work less than 20 h per week, for a substantially higher income.

Interestingly, our results suggest little influence of private or public school training in physicians’ choices of practice sector. Previous studies in Brazil have shown that the public sector is responsible for providing specialist titles for a large proportion of the medical workforce, and that most specialists deliver services through private services [24]. Our findings seem to support scholars’ opinion that the public education may not necessarily be instrumental for retaining specialists in the public services [25].

Although the cross-sectional model used in this study mapped out the current profile of physicians working in both public and private sectors, it has limited power to predict the trajectory of physician’s careers over time; still, some preliminary conclusions can be drawn. It appears that younger and less experienced physicians are concentrated among doctors delivering public services, mostly at primary care units, public hospitals and on-call services; this could be partially explained by the fact that most internship programs are provided by public universities and hospitals, especially at primary care and general practice areas. Public practitioners work mostly 20 to 40 h per week, and often have to commute between job and their residence location. Moreover, these job positions frequently provide lower remuneration when compared to private working positions and are frequently located at poor peripheral areas; such conditions may impact on the residence decisions of more experienced physicians in public services. As doctors become more experienced and specialised, they may start migrating towards those urban areas offering ampler opportunities for private practice, dedicating only part-time jobs to public services [26]. These practitioners have higher income than those physicians dedicated exclusively to the public practice, but are also subjected to longer working hours to keep both job positions. Private practitioners engage exclusively with medical services in private hospital institutions or at their own medical offices, and working exclusively as private practitioner appears to provide access to higher incomes; according to the income-leisure trade-off theory [27, 28] this would allow physicians to effectively reduce their weekly workloads, which would explain the over-representation of such physicians in the 20 h per week category. Obviously, older physicians may be working shorter hours in Brazil because they are no longer able to work full-time, but an alternative explanation could be that they are already established and wealthy enough to dedicate more time to leisure activities.

Such patterns are consistent with the existing worldwide literature on this subject [29, 30], and the dual practice prevalence described in this study is not dissimilar from those found in low and middle-income countries, such as Peru [31], Indonesia [32, 33], Thailand [34], Mexico, Egypt and Kenya [35], where the majority of physicians also seemed to engage in multiple job-holding. Despite the peculiarities of its health sector, Brazil’s findings appear to confirm that some contextual factors like governance and economic growth play a primary role in dual practice prevalence and forms [12, 36]. Demand for services, opportunities for private sector engagement, and how clearly defined public and private systems are configured seem to be key determinants of the frequency and forms in which physicians take on simultaneous profit generating activities in the two sectors. Perhaps differently from what observed in other contexts, the Brazilian case shows a more marked trend of public sector physicians’ engagement with private services, starting with the younger age cohorts.

Some authors highlighted their concern that physicians’ dual practice may reduce the accessibility and/or quality of care available to users of the public system [3, 37, 38]. As in Brazil prepaid and private out-of-pocket expenditures represents 54% of the total health spending [17], our findings lend credibility to the hypothesis that physicians’ migration to the private sector may pose challenges to population access to medical services. Previous analyses tackling inequalities of the Brazilian health system have identified negative impacts of the private sector over public-based health services, such as the shortage of public services, decreased funding of human resources and the decreased governmental investments on the SUS [17, 39].

The present study has shown that more than 78% of physicians are engaged in private services (including dual practitioners), whereas 73.1% provide healthcare by state-financed healthcare structures (also including dual practitioners). Considering the part-time dedication of dual practitioners, these results reinforces concerns of reduced access to services, once the population dependent solely on public care represents three fold the population of private plan users [40]. Furthermore, other studies [23] compared the number of working positions across the public and private sectors in Brazil over the years 2002, 2005 and 2009, revealing an increased concentration of working positions in private services when compared to working positions of the SUS, despite the continuous growth of the medical population in Brazil. Based on this scenario characterized by the absence of proper dual practice regulation and a growing tendency of physicians to take job positions at private services, the inauguration of new medical schools and medical residence programs predicted by the More Doctors Law may not yield positive effects over the distribution of medical workforce to cover the poorest and under-assisted regions, which are still highly dependent on public health services.

## Conclusion

The present study used original primary data to describe the profile of physicians currently working in Brazil, one of world’s largest countries, with one of the world’s most complex health systems.

Our findings highlight substantial differences in the proportion and socio-demographic/work-related characteristics between physicians engaging in public and private services, with a specific reference to those engaging in dual practice. As the vast majority of Brazilian citizens are highly dependent on public healthcare services, the high proportion of physicians working as dual and private practitioners found in this study may raise doubts for population’s access to public services. This scenario demands attention from public health authorities and further exploration of the motivations behind such behaviour in Brazil is needed to support policies addressing the maldistribution of physicians across public and private services.

This study draws from the findings of the country’s first medical demography study, and we highlight the need for further analysis to be carried out to evaluate the impact of dual practice on the provision of services. By comparing and contrasting the Brazilian physicians’ case with other country experiences, the present study enriches the existing literature and the ongoing debate on this subject.

## Additional files

Additional file 1: Questionnaire used to obtain primary data. Additional file 1 shows the full version of the questionnaire used by the interviewers to obtain primary data for this study. (DOCX 79 kb)

Additional file 2: Sociodemographic profile of Brazilian physicians according to their public/dual/private modality of practice. Additional file 2 shows all prevalence rates and confidence intervals obtained from the socioeconomic variables included in this study. (DOCX 88 kb)

Additional file 3: Medical work characteristics of Brazilian physicians according to their public/dual/private modality of practice. Additional file 3 shows all prevalence rates and confidence intervals obtained from the medical work related variables included in this study. (DOCX 126 kb)

## Abbreviations

CFM: Conselho Federal de Medicina
CI: Confidence intervals
n: Number
PR: Prevalence Ratios
SPSS: Statistical Package for the Social Sciences
SUS: Sistema Único de Saúde
