# Supplementary material for: Physician’s sociodemographic profile and distribution across public and private health care: an insight into physicians’ dual practice in Brazil
Source: BMC Health Serv Res. 2018 Apr 23;18:299. doi: 10.1186/s12913-018-3076-z (PMC5914025; doi:10.1186/s12913-018-3076-z)
Supplement: Supplementary file 1 — Questionnaire used to obtain primary data. Additional file 1 shows the full version of the questionnaire used by the interviewers to obtain primary data for this study. (DOCX 79 kb) [file 12913_2018_3076_MOESM1_ESM.docx]

**Additional File 1: Questionnaire used to obtain primary data**

**Q.A** Sex: 1. Masculine 2. Feminine

**Q.B** What is your age? **(ESPONTANEOUS AND PONCTUAL)**

|  | WRITE DOWN |
| --- | --- |

**Q.1** I will read four professional academic situations and would like to know in which of the following situations better describe yourself? **(READ CALMLY UNTILL THE EXCLAMATION MARK – STIMULATED AND PONCTUAL)**

1. Specialist with Specialty Society or Medical Internship degree (even if it’s still in progress);
2. Physician without specialty or Medical Internship degree, experienced in a specific medical field;
3. Physician without specialty or Medical Internship degree, practicing medicine in two or more medical fields; **OR**
4. Physician that works primarily in a non-clinical/diagnostic/cirurgical field**?**

**Q.1A (FOR P1=2)** which area? ____________________________________________________

**P.1B** **(FOR P1=4)** what field? ______________________________________

**Q.2 (FOR ALL)** You **currently** work ________ (**CITE EACH ITEM FROM TABLE)?** In another clinical or non-clinical area not previously mentioned? **(STIMUATED AND PONCTUAL FOR EACH LINE)**

**Q.3 (FOR EACH YES FROM Q.2)** You **currently** work _________________ in the private or public sector?

**STIMUATED AND PONCTUAL FOR EACH LINE)**

|  | **Q2 Practice medicine** | | **Q3 Sector** | | |
| --- | --- | --- | --- | --- | --- |
|  | **Yes** | **No** | **Private** | **Public** | **Both** |
| **CLINICAL FIELD** |  |  |  |  |  |
| a. with clinics? ( medical care/consultation ) | 1 | 2 | 1 | 2 | 3 |
| b. with diagnostics? ( performs exams ) | 1 | 2 | 1 | 2 | 3 |
| c. with surgery ? ( performs surgeries ) | 1 | 2 | 1 | 2 | 3 |
| **NON-CLINICAL FIELD** |  |  |  |  |  |
| d. in Management, Direction, Administration of services*? | 1 | 2 | 1 | 2 | 3 |
| f. teaching? | 1 | 2 | 1 | 2 | 3 |
| g. with research? | 1 | 2 | 1 | 2 | 3 |
| 98. Other **(WRITE DOWN)** ____________ | 1 | 2 | 1 | 2 | 3 |

*** RESEARCHER: IF THE INTERVIEWEE MENTIONS THAT “MANAGES A CLINICAL SERVICE OU HIS OWN PRACTICE OFFICE” 🡪 CONSIDER Q2s = 2**

**Q.4** You work as a physician in a full-time or a part-time job? **(PONCTUAL)**

1. Full-time 2. Part-time

**FOR THOSE WORKING IN THE PRIVATE SECTOR Q3=1 or Q3=3**

**Q.5** You have told me that you work in the private sector. You **currently** work in: ________________. (**READ THE OPTIONS FROM THE TABLE BELOW)** Any other private segment not previously mentioned?

**Q.6 (FOR EACH YES FROM Q5)** How many jobs/employments do you have in__________ **(READ EACH WORKPLACE)**

**Q.7 (FOR EACH YES FROM Q5)** What is your workload in a typical week in_________ (**READ EACH WORKPLACE)
CHECK:** Is this a weekly workload?

**Q.8 (FOR EACH YES FROM Q5)** What kind of hiring contract that you have in ________________ (**READ EACH WORK PLACE):** CLT; CNPJ; Cooperative; Autonomous/RPA or any other category not previously mentioned? **(ONE ANSWER PER LINE)**

**Q.9 (FOR EACH YES FROM Q5)** How many patients do you provide medical care in a typical day in __________ **(READ EACH WORKPLACE)?
CHECK:** Is this the quantity of patients that you provide daily medical care? **(ESPONTANEOUS AND PONCTUAL)**

|  | **Q.5 Workplace** | | **Q.6**  **How many jobs?** | **Q.7**  **Week workload** | **Q.8**  **Hiring contract** | | | | | **Q.9 Nº patients attended** |
| --- | --- | --- | --- | --- | --- | --- | --- | --- | --- | --- |
|  | **Yes** | **No** |  | **In hours** | CLT | CNPJ | Cooperative | Autonomous/ RPA | Other | **Per day** |
| 1. Private hospital? | 1 | 2 |  | ­­­­­­­­­­­ | 1 | 2 | 3 | 4 | 98_______ |  |
|  |  |  |  |  | 1 | 2 | 3 | 4 | 98_______ |  |
| 1. Private service/ambulatory? | 1 | 2 |  |  | 1 | 2 | 3 | 4 | 98_______ |  |
| 1. Private practice office? | 1 | 2 |  |  | 1 | 2 | 3 | 4 | 98_______ |  |
|  | 1 | 2 |  |  | 1 | 2 | 3 | 4 | 98_______ |  |
| 1. Private diagnostic laboratory? | 1 | 2 |  |  | 1 | 2 | 3 | 4 | 98_______ |  |
| 1. Pharmaceutical industry? | 1 | 2 |  |  | 1 | 2 | 3 | 4 | 98_______ |  |
| 1. Private company physician? | 1 | 2 |  |  | 1 | 2 | 3 | 4 | 98_______ |  |
| 1. Private university? | 1 | 2 |  |  | 1 | 2 | 3 | 4 | 98_______ |  |
| 1. Other private workplace? **(WRITE DOWN)** __________ | 1 | 2 |  |  | 1 | 2 | 3 | 4 | 98_______ |  |

**FOR THOSE WORKING IN A PRIVATE PRACTICE OFFICE and/or PRIVATE CLINICAL SERVICE/AMBULATORY**

**Q5c =1 AND/OR Q5b=1**

**Q.10** Are you the owner of the private office or private clinical service/ambulatory or are you a service provider?

1. Owner/Autonomous/Liberal **(MOVE ON)** 2. Service provision/employee **🡪 JUMP TO Q.10B** 3. Both

**Q.10A (IF OWNS A PRIVATE OFFICE/PRIVATE CLINICAL SERVICE AS AN AUTONOMOUS/LIBERAL OR BOTH,**

**~~P10=1 or 3~~) When did you started the office/clinical service? (SPONTANEOUS AND PONCTUAL)**

|  | WRITE DOWN THE YEAR |
| --- | --- |

**Q.11**  Do you currently work in a private office or clinical service: _______ **(READ UNTIL THE EXCLAMATION MARK – STIMULATED AND MULTIPLE ASWERS ALLOWED)**

1. Alone,
2. Share office/clinical service with another professional **OR**
3. Share office/clinical service with many professionals**?**

98. Other **(WRITE DOWN)** _____________________________________________

**Q.12** Currently in your office/clinical service, do you provide medical care to: **(READ EACH ITEM – STIMULATED AND PONCTUAL)**

**Q.13** **(FOR Q12=1)** Approximately how many hours do you spend providing medical care to private users? What about patients covered by healthcare plans/insurance companies? **(STIMULATED AND ONE ANSWER PER LINE)**

**CHECK, ASKING**: Is this a weekly workload?

**Q.14** What is the percentage of your incomes from the office or clinical service come from: __ **(READ OPTIONS BELOW) ATTENTION: TOTAL SHOULD BE COMPUTATED AS 100%**

|  | **Q.12 Provides** | | **Q.13 Nº hours worked weekly** | **Q.14 % incomes** |
| --- | --- | --- | --- | --- |
|  | **Yes** | **No** |  |  |
| 1. Private patients? | 1 | 2 | hours |  |
| 1. patients covered by healthcare plans/insurance companies | 1 | 2 | hours |  |
|  |  |  |  | **TOTAL: 100%** |

**Q. 10B (Q5a; d; e; f; g or h=1 and/or Q10 = 2 )** Have you worked in this private practice office/clinical service as an autonomous/liberal professional, instead of as a service provider? **(PONCTUAL)**

1. Yes 2. No

**FOR THOSE WORKING IN THE PUBLIC SECTOR Q3=2 or Q3=3**

q

**Q.15** You have told me that you work in the public sector. You **currently** work in: _______________ (**READ THE OPTIONS FROM THE TABLE BELOW)** Any other public segment not previously mentioned?

**Q.16 (FOR EACH YES FROM Q15)** How many jobs/employments do you have in__________ **(READ EACH WORKPLACE)**

**Q.17 ((FOR EACH YES FROM Q15)** What is your workload in a typical week in_________ (**READ EACH WORKPLACE)
CHECK:** Is this a weekly workload?

**Q.18 (FOR EACH YES FROM Q15)** Who is your employer: Direct administration~~:~~ (prefecture, state government or federal government); Social Organization; Foundation, Autarchy or any category not previously mentioned**?** **(STIMULATED AND MULTIPLE ASWERS ALLOWED)**

**Q18A** **(FOR EACH YES FROM Q15)** What kind of hiring contract that you have in ______**(READ EACH WORKPLACE):** State contracted employee (municipal, state or federal government); CLT; CNPJ; Cooperative, Autonomous/RPA or any category not previously mentioned**?** **(ONE ANSWER PER LINE)
- IF ANSWERS “STATE CONTRACT”, ASK:** State contracted employee or CLT?
**- IF ANSWERS “NO CONTRACT”, ASK:** CLT, CNPJ or RPA?

**Q.19 (FOR EACH YES FROM Q15)** How many patients do you provide medical care in a typical day in __________ **(READ EACH WORKPLACE)?
CHECK:** Is this the quantity of patients that you provide daily medical care?

|  | **Q.15**  **Workplace** | | **Q.16**  **How many jobs?** | **Q.17 Week workload** | **Q.18**  **Employer** | | | | | **Q.18A**  **Hiring contract** | | | | | | **Q.19**  **Nº patients attended (daily)** |
| --- | --- | --- | --- | --- | --- | --- | --- | --- | --- | --- | --- | --- | --- | --- | --- | --- |
|  | **Yes** | **No** |  |  | Direct Adm. | S.O. | Foundation | Autarchy | Other | Contracted | CLT | CNPJ | Cooperative | Auton./RPA | Other |  |
| 1. University hospital? | 1 | 2 |  |  | 1 | 2 | 3 | 4 | 98____ | 1 | 2 | 3 | 4 | 5 | 98______ |  |
|  |  |  |  |  | 1 | 2 | 3 | 4 | 98____ | 1 | 2 | 3 | 4 | 5 | 98______ |  |
| 1. “Santa Casa” or Philanthropic Hospital? | 1 | 2 |  |  | 1 | 2 | 3 | 4 | 98____ | 1 | 2 | 3 | 4 | 5 | 98______ |  |
| 1. Other Public Hospital? | 1 | 2 |  |  | 1 | 2 | 3 | 4 | 98____ | 1 | 2 | 3 | 4 | 5 | 98______ |  |
|  | 1 | 2 |  |  | 1 | 2 | 3 | 4 | 98____ | 1 | 2 | 3 | 4 | 5 | 98______ |  |
| 1. Primary care? | 1 | 2 |  |  | 1 | 2 | 3 | 4 | 98____ | 1 | 2 | 3 | 4 | 5 | 98______ |  |
| 1. Public University? | 1 | 2 |  |  | 1 | 2 | 3 | 4 | 98____ | 1 | 2 | 3 | 4 | 5 | 98______ |  |
| 1. Other Public Facility? **(WRITE DOWN)** __________ | 1 | 2 |  |  | 1 | 2 | 3 | 4 | 98____ | 1 | 2 | 3 | 4 | 5 | 98______ |  |

**Q.20**  **(FOR THOSE WORKING IN A UNIVERSITY HOSPITAL, “SANTA CASA”/ PHYLANTROPIC OR PUBLIC HOSPITAL, Q15a = 1, Q15b = 1, Q15c = 1)** In what hospital area do you work: Ambulatory, Intensive care/surgical centre or Primary care/Emergency? **(STIMULATED AND MULTIPLE ASWERS ALLOWED)**

1. Ambulatory
2. Intensive care/ surgical centre OR
3. Primary care/Emergency?

**Q.21** **(FOR THOSE WORKING AT THE PRIMARY CARE NETWORK, Q15d = 1)** Do you work at a Primary Care Unit, Health Care Centre, Health Care Unit or Family Health Care? **(STIMULATED AND MULTIPLE ASWERS ALLOWED)**

1. Primary Care Unit (UBS)
2. Health Care Centre
3. Health Care Unit OR
4. Family Health Care?

**APPLY TO ALL**

**Q.23 In general, you would say that, currently**: **(READ UNTILL THE EXCLAMATION MARK – STIMULATED AND PONCTUAL)**

1. You are facing a work overload,

2. You are working at full capacity **OR**

3. You could work more**?**

**Q.24** Approximately how many 24-hour shifts do you work weekly? **(SPONTANEOUS AND PONCTUAL)**

|  |  |
| --- | --- |

96. Doesn’t work 24-hour shifts **🡪 JUMP TO Q.26**

**Q.25** **(IF WOKS 24-HOUR SHIFTS, Q24) How many hour do you work each 24-hour shift? (SPONTANEOUS)**

How many shifts How many hours

**RESEARCHER: CHECK THE NUMBER OF SHIFTS BETWEEN Q.24 AND Q.25**

**Q.26** What is your main income category considering all income resources acquired from the medical profession______ **(READ THE OPTIONS BELOW – STIMULATED AND PONCTUAL)**

1. Monthly compensation,
2. Working hour compensation,
3. Compensation depends on the number of procedures performed,
4. Compensation depends on the number of patients attended **OR**
5. Compensation depends on the number of groups of patients attended**?**

98. Other **(WRITE DOWN)** ______________________________________________________________

**Q.27** I will read some income stratus and would like to know which of then describes better your situation. Considering all job categories related to the medical profession that you practice, approximately how much money do you make per month? **(STIMUATED AND PONCTUAL)**

1. Up to R$8.000
2. From R$8.001 to R$12.000
3. From R$12.001 to R$16.000
4. From R$16.001 to R$20.000
5. R$20.001 to R$24.000
6. R$24.001 or more

97. Refuses to say

**Q.28** Are your multiple workplaces located: _____ **(READ UNTILL THE EXCLAMATION MARK – STIMULATED AND PONCTUAL)**

1. In the same city that you live 2. In a different city OR 3. Partly in the same city and e partly in another city?

**Q.29** How many kilometres do you dislocate between you job and work (and vice-versa) in a typical week: ______________ **(READ THE INTERVALS BELOW UNTILL THE EXCLAMATION MARK) CHECK ASKING**: Does this number represents how many kilometres you dislocate weekly? **(STIMULATED)**

1. Up to 25 km,

2. From 26 to 50 km,

3. From 51 to 100 km,

4. From101 to 250 km

5. 251 a 400 km **OR**

6. 401 km or more **?**

### Q.30 I will quote seven aspects that might fixate a physician in a workplace or make him/her chance to another place. I would like for you to ordinate these topics going from the most important to the least important, from the 1º to the 7º position. _________ (READ THE ITENS BELOW – ONE PER COLUMM)

**RESEARCHER:**

**1º REAR ALL ITENS**

**2º ASK:** Which of these do you consider the first and more important item to fixate a physician in his/her workplace? And in second place? And in third place? …………..

| **ATTENTION FOR THE ROTATION** | **1º PLACE** | **2º PLACE** | **3º PLACE** | **4º PLACE** | **5º PLACE** | **6º PLACE** | **7º**  **PLACE** |
| --- | --- | --- | --- | --- | --- | --- | --- |
| 1. Work conditions | 1 | 1 | 1 | 1 | 1 | 1 | 1 |
| 1. Wage, remuneration | 2 | 2 | 2 | 2 | 2 | 2 | 2 |
| 1. Possibility of improving skills and specialization | 3 | 3 | 3 | 3 | 3 | 3 | 3 |
| 1. Secure and non-violent workplace | 4 | 4 | 4 | 4 | 4 | 4 | 4 |
| 1. Professional appreciation | 5 | 5 | 5 | 5 | 5 | 5 | 5 |
| 1. Career plan | 6 | 6 | 6 | 6 | 6 | 6 | 6 |
| 1. Life quality | 7 | 7 | 7 | 7 | 7 | 7 | 7 |
| 1. None | //// | 96 | 96 | 96 | 96 | 96 | 96 |

**Q.22** To conclude, in case of wage, working conditions and hours worked differences between the public and private sector did not exist, would you work in the public or in the private sector? **(STIMULATED AND PONCTUAL)**

1. Public sector 2. Private sector

### AKNOWLEGDE AND STOP THE QUESTIONAIRE

|  | |  |  |
| --- | --- | --- | --- |
|  |  |  |  |
|  | |  |  |
|  | |  |  |
|  | |  |  |
| - | |  |  |
|  | |  |  |
|  | |  |  |
|  | |  |  |
|  | |  |  |
|  | |  |  |
|  | |  |  |
|  | |  |  |
|  | |  |  |
|  | |  |  |
|  | |  |  |
|  | |  |  |
|  | |  |  |
|  | | | |
