# Supplementary material for: Physician’s sociodemographic profile and distribution across public and private health care: an insight into physicians’ dual practice in Brazil
Source: BMC Health Serv Res. 2018 Apr 23;18:299. doi: 10.1186/s12913-018-3076-z (PMC5914025; doi:10.1186/s12913-018-3076-z)
Supplement: Supplementary file 2 — Sociodemographic profile of Brazilian physicians according to their public/dual/private modality of practice. Additional file 2 shows all prevalence rates and confidence intervals obtained from the socioeconomic variables included in this study. (DOCX 88 kb) [file 12913_2018_3076_MOESM2_ESM.docx]

**Additional File 2:** Sociodemographic profile of Brazilian physicians according to their public/dual/private modality of practice.

| **Sociodemographic characteristics** | **Public practice** | | **Dual practice** | | **Private practice** | | **Total** | |
| --- | --- | --- | --- | --- | --- | --- | --- | --- |
|  | **N** | **% (95% CI)** | **N** | **% (95% CI)** | **N** | **% (95% CI)** | **N** | **% (95% CI)** |
| *Gender* |  |  |  |  |  |  |  |  |
| Female | 273 | 52.7 (48.6-56.9) | 515 | 41.7 (38.9-44.5) | 233 | 36.0 (32.6-39.6) | 1021 | 42.5 (40.4-44.6) |
| Male | 245 | 47.3 (43.1-51.4) | 720 | 58.3 (55.5-61.1) | 414 | 64.0 (60.4-67.4) | 1379 | 57.5 (55.4-59.6) |
| **Total** | **518** | **-** | **1235** | **-** | **647** | **-** | **2400** | **-** |
| *Age (years)* |  |  |  |  |  |  |  |  |
| < 35 | 208 | 40.2 (35.9-44.4) | 415 | 33.6 (30.9-36.4) | 121 | 18.7 (16.1-21.8) | 744 | 31.0 (29.1-32.9) |
| 35 - 60 | 220 | 42.5 (38.2-46.5) | 681 | 55.1 (52.4-58.0) | 306 | 47.3 (43.4-51.3) | 1207 | 50.3 (48.3-52.3) |
| > 60 | 90 | 17.4 (14.1-20.7) | 139 | 11.3 (9.5-13.1) | 220 | 34.0 (30.4-37.9) | 449 | 18.7 (17.1-20.2) |
| **Total** | **518** | **-** | **1235** | **-** | **647** | **-** | **2400** | **-** |
| *Brazilian region* |  |  |  |  |  |  |  |  |
| Northern | 22 | 4.2 (2.5-6.2) | 65 | 5.3 (4.0-6.6) | 17 | 2.6 (1.5-3.9) | 104 | 4.3 (3.4-5.2) |
| Northeastern | 101 | 19.5 (16.2-23.2) | 240 | 19.4 (17.3-21.7) | 73 | 11.3 (9.0-13.8) | 414 | 17.3 (15.7-18.7) |
| Southeastern | 299 | 57.7 (52.9-62.0) | 666 | 53.9 (51.0-57.0) | 380 | 58.7 (55.0-62.3) | 1345 | 56.0 (54.0-58.0) |
| Southern | 71 | 13.7 (10.8-16.8) | 169 | 13.7 (11.7-15.6) | 114 | 17.6 (14.7-20.6) | 354 | 14.8 (13.5-16.2) |
| Center-western | 25 | 4.8 (2.9-6.8) | 95 | 7.7 (6.2-9.1) | 63 | 9.7 (7.4-12.2) | 183 | 7.6 (6.6-8.7) |
| **Total** | **518** | **-** | **1235** | **-** | **647** | **-** | **2400** | **-** |
| *Local of address* |  |  |  |  |  |  |  |  |
| Capital | 291 | 56.2 (51.9-60.6) | 669 | 54.2 (51.5-57.1) | 391 | 60.4 (56.7-64.5) | 1351 | 56.3 (54.3-58.1) |
| Countryside | 227 | 43.8 (39.4-48.1) | 566 | 45.8 (42.9-48.5) | 256 | 39.6 (35.5-43.3) | 1049 | 43.7 (41.9-45.7) |
| **Total** | **518** | **-** | **1235** | **-** | **647** | **-** | **2400** | **-** |
| *Medical education* |  |  |  |  |  |  |  |  |
| Public | 316 | 63.6 (59.2-68.0) | 763 | 63.4 (60.6-65.9) | 415 | 65.7 (62.0-69.3) | 1494 | 64.0 (62.1-66.0) |
| Private | 181 | 36.4 (32.0-40.8) | 441 | 36.6 (34.1-39.4) | 217 | 34.3 (30.7-38.0) | 839 | 36.0 (34.0-37.9) |
| **Total** | **497** | **-** | **1204** | **-** | **632** | **-** | **2333** | **-** |
