# Supplementary material for: Physician’s sociodemographic profile and distribution across public and private health care: an insight into physicians’ dual practice in Brazil
Source: BMC Health Serv Res. 2018 Apr 23;18:299. doi: 10.1186/s12913-018-3076-z (PMC5914025; doi:10.1186/s12913-018-3076-z)
Supplement: Supplementary file 3 — Medical work characteristics of Brazilian physicians according to their public/dual/private modality of practice. Additional file 3 shows all prevalence rates and confidence intervals obtained from the medical work related variables included in this study. (DOCX 126 kb) [file 12913_2018_3076_MOESM3_ESM.docx]

**Additional File 3:** Medical work characteristics of Brazilian physicians according to their public/dual/private modality of practice.

| **Medical Work Characteristics** | **Public practice** | | **Dual practice** | | **Private practice** | | **Total** | |
| --- | --- | --- | --- | --- | --- | --- | --- | --- |
|  | **N** | **% (95% CI)** | **N** | **% (95% CI)** | **N** | **% (95% CI)** | **N** | **% (95% CI)** |
| *Job X residence location* |  |  |  |  |  |  |  |  |
| Same city | 317 | 61.2 (56.8-65.6) | 712 | 57.7 (55.1-60.4) | 509 | 78.7 (75.6-81.6) | 1538 | 64.1 (62.1-65.9) |
| Different cities | 84 | 16.2 (12.9-19.5) | 57 | 4.6 (3.4-5.8) | 28 | 4.3 (2.8-5.9) | 169 | 7.0 (6.1-8.1) |
| Both | 117 | 22.6 (19.1-26.3) | 466 | 37.7 (34.9-40.5) | 110 | 17.0 (14.4-19.8) | 693 | 28.9 (27.1-30.8) |
| **Total** | **518** | **-** | **1235** | **-** | **647** | **-** | **2400** | **-** |
| *Work affiliation* |  |  |  |  |  |  |  |  |
| *Private assistance* |  |  |  |  |  |  |  |  |
| Clinic/ambulatory | - | - | 486 | 39.4 (36.5-42.1) | 260 | 40.2 (36.5-44.2) | 746 | 31.1 (29.3-32.8) |
| Physician's Office | - | - | 612 | 49.6 (46.8-52.7) | 351 | 54.3 (50.5-58.4) | 963 | 40.1 (38.1-42.1) |
| Hospital | - | - | 658 | 53.3 (50.4-56.0) | 256 | 39.6 (36.2-43.4) | 914 | 38.1 (36.2-40.1) |
| University/College | - | - | 96 | 7.8 (6.3-9.3) | 31 | 4.8 (3.1-6.5) | 127 | 5.3 (4.3-5.2) |
| *Public assistance* |  |  |  |  |  |  |  |  |
| Hospital | 353 | 68.1 (63.9-72.0) | 883 | 71.5 (68.9-73.8) | - | - | 1236 | 51.5 (49.5-53.5) |
| Primary health care | 214 | 41.3 (37.1-45.7) | 349 | 28.3 (25.8-30.9) | - | - | 563 | 23.5 (22-25.2) |
| Specialized health care | 33 | 6.4 (4.2-8.5) | 82 | 6.6 (5.3-8.0) | - | - | 115 | 4.8 (3.9-5.7) |
| University/College | 30 | 5.8 (3.9-7.9) | 69 | 5.6 (4.4-6.9) | - | - | 99 | 4.1 (3.3-5.0) |
| *On-call services* |  |  |  |  |  |  |  |  |
| Yes | 295 | 56.9 (52.7-61) | 678 | 54.9 (52.4-57.5) | 118 | 18.2 (15.3-21.2) | 1091 | 45.5 (43.5-47.5) |
| No | 223 | 43.1 (39.0-47.3) | 557 | 45.1 (42.5-47.6) | 529 | 81.8 (78.8-84.7) | 1309 | 54.5 (52.5-56.5) |
| **Total** | **518** | **-** | **1235** | **-** | **647** | **-** | **2400** | **-** |
| *Years of Medical Practice*** |  |  |  |  |  |  |  |  |
| < 10 | 203 | 40.1 (35.8-44.3) | 382 | 31.3 (28.6-34.0) | 113 | 17.6 (14.6-20.4) | 698 | 29.5 (27.5-31.4) |
| 10 --\| 30 | 158 | 31.2 (27.1-35.4) | 567 | 46.5 (43.6-49.3) | 237 | 36.9 (33.2-40.5) | 962 | 40.6 (38.6-42.6) |
| > 30 | 145 | 28.7 (24.5-32.6) | 270 | 22.1 (19.9-24.5) | 292 | 45.5 (41.7-49.5) | 707 | 29.9 (28.0-31.8) |
| **Total** | **506** | **-** | **1219** | **-** | **642** | **-** | **2367** | **-** |
| *Weekly workload (hours)* |  |  |  |  |  |  |  |  |
| < 20 | 35 | 6.8 (4.6-9.1) | - | - | 89 | 13.8 (11.3-16.4) | 124 | 5.2 (4.3-6.2) |
| 20 --\| 40 | 176 | 34.0 (29.7-37.8) | 80 | 6.5 (5.0-7.9) | 209 | 32.3 (28.9-35.9) | 465 | 19.4 (18.0-20.9) |
| 40 --\| 60 | 204 | 39.4 (35.5-43.4) | 569 | 46.1 (43.2-48.8) | 261 | 40.3 (36.8-44.0) | 1034 | 43.1 (41.1-45.0) |
| > 60 | 103 | 19.9 (16.6-23.6) | 586 | 47.4 (44.5-50.3) | 88 | 13.6 (11.0-16.2) | 777 | 32.4 (30.5-34.2) |
| **Total** | **518** | **-** | **1235** | **-** | **647** | **-** | **2400** | **-** |
| *Medical specialty* |  |  |  |  |  |  |  |  |
| Clinic | 174 | 33.6 (29.7-37.6) | 410 | 33.2 (30.4-35.9) | 219 | 33.8 (30.4 - 37.4) | 803 | 33.5 (31.6-35.3) |
| Surgery | 50 | 9.7 (7.1-12.4) | 220 | 17.8 (15.6-19.9) | 104 | 16.1 (13.1 - 18.9) | 374 | 15.6 (14.0-17.1) |
| Both | 49 | 9.5 (7.1-12.0) | 287 | 23.2 (20.8-25.7) | 117 | 18.1 (15.3 - 21.2) | 453 | 18.9 (17.3-20.5) |
| No specialty | 245 | 47.3 (43.1-51.4) | 318 | 25.7 (23.2-28.3) | 207 | 32.0 (28.4 - 35.5) | 770 | 32.1 (30.3-34.0) |
| **Total** | **518** | **-** | **1235** | **-** | **647** | **-** | **2400** | **-** |
| *Income (per month)* |  |  |  |  |  |  |  |  |
| ≤ US$3.857.00 | 196 | 37.8 (33.6-41.9) | 143 | 11.6 (10.0-13.4) | 141 | 21.8 (18.9-25.2) | 480 | 20 (18.4-21.7) |
| US$3.857.00 --\| 5.381.00 | 135 | 26.1 (22.2-30.1) | 293 | 23.7 (21.1-26.1) | 106 | 16.4 (13.8-19.3) | 534 | 22.3 (20.6-23.8) |
| US$5.381.00 --\| 7.175.00 | 94 | 18.1 (14.9-21.4) | 277 | 22.4 (20.0-24.8) | 111 | 17.2 (14.4-20.1) | 482 | 20.1 (18.6-21.7) |
| US$7.175.00 --\| 8.969.00 | 49 | 9.5 (6.9-12.2) | 187 | 15.1 (12.8-17.2) | 79 | 12.2 (9.7-14.7) | 315 | 13.1 (11.8-14.5) |
| US$8.969.00 --\| 10.762.00 | 20 | 3.9 (2.1-5.6) | 103 | 8.3 (6.9-9.8) | 52 | 8.0 (6.0-10.2) | 175 | 7.3 (6.3-8.4) |
| ≥ US$10.762.00 | 12 | 2.3 (1.0-3.7) | 189 | 15.3 (13.4-17.2) | 121 | 18.7 (15.9-21.8) | 322 | 13.4 (12.0-14.7) |
| Did not answer | 12 | 2.3 (1.0-3.9) | 43 | 3.5 (2.5-4.6) | 37 | 5.7 (4.0-7.6) | 92 | 3.8 (3.0-4.6) |
| **Total** | **518** | **-** | **1235** | **-** | **647** | **-** | **2400** | **-** |
| ** Missing data = 33 |  |  |  |  |  |  |  |  |
